# Uncontrolled sepsis: a systematic review of translational immunology studies in intensive care medicine

**DOI:** 10.1186/2197-425X-2-6

**Published:** 2014-02-27

**Authors:** David J Cain, Ana Gutierrez del Arroyo, Gareth L Ackland

**Affiliations:** Clinical Physiology, Wolfson Institute for Biomedical Research, Department of Medicine, University College London, London, WC1E 6BT UK

**Keywords:** Sepsis, Immunology, Human, Critical care, Surgical intensive care

## Abstract

**Background:**

The design of clinical immunology studies in sepsis presents several fundamental challenges to improving the translational understanding of pathologic mechanisms. We undertook a systematic review of bed-to-benchside studies to test the hypothesis that variable clinical design methodologies used to investigate immunologic function in sepsis contribute to apparently conflicting laboratory data, and identify potential alternatives that overcome various obstacles to improve experimental design.

**Methods:**

We performed a systematic review of the design methodology employed to study neutrophil function (respiratory burst), monocyte endotoxin tolerance and lymphocyte apoptosis in the intensive care setting, over the past 15 years. We specifically focussed on how control samples were defined, taking into account age, gender, ethnicity, concomitant therapies, timing of sample collection and the criteria used to diagnose sepsis.

**Results:**

We identified 57 eligible studies, the majority of which (74%) used case–control methodology. Healthy volunteers represented the control population selected in 83% of studies. Comprehensive demographic data on age, gender and ethnicity were provided in ≤48% of case control studies. Documentation of diseases associated with immunosuppression, malignancy and immunomodulatory therapies was rare. Less than half (44%) of studies undertook independent adjudication for the diagnosis of sepsis while 68% provided microbiological data. The timing of sample collection was defined by highly variable clinical criteria. By contrast, surgical studies avoided many such confounders, although only one study in surgical patients monitored the study group for development of sepsis.

**Conclusions:**

We found several important and common limitations in the clinical design of translational immunologic studies in human sepsis. Major elective surgery overcame many of these methodological limitations. The failure of adequate clinical design in mechanistic studies may contribute to the lack of translational therapeutic progress in intensive care medicine.

## Background

Mortality from sepsis is persistently high, and may even be rising despite decades of research [1, 2]. Promising pre-clinical immunomodulatory therapies have failed in clinical practice [3–5] perhaps attributable, in part, to differences between human and rodent immunology [6]. However, an alternative explanation is that the heterogeneous etiology, presentation and progression of human sepsis generate confounding factors that distort the interpretation of clinical immunologic studies. Thus, the identification of appropriate controls, diagnostic accuracy, demographic influences and therapies with immunomodulatory off-target effects are critical considerations in interpreting translational work.

We therefore systematically reviewed the clinical experimental design of studies in three key areas of bed-to-benchside immunologic research in sepsis, focusing in particular on comparator groups and the documentation of known confounding factors. We also explored how the investigation of immune mechanisms in other clinical scenarios - trauma and major elective surgery - associated with the development of sepsis may help refine experimental design.

## Methods

A Pubmed search was performed for the terms‘Neutrophil respiratory burst’ OR‘Monocyte endotoxin tolerance’ OR‘Lymphocyte apoptosis’ AND‘Sepsis’ OR‘Trauma’ OR‘Surgery’, restricted to adult human studies published between 03 January 1998 and 03 January 2013. The abstract of each paper was manually assessed for suitability. *In vitro* studies of healthy volunteer cells were excluded.

### Clinical demographics

For all eligible manuscripts, we recorded the primary author, year of publication and clinical setting. The number, age, gender, clinical severity score of subjects and their corresponding controls, in whom the same assay of immune function was performed, were compared. The criteria used to define sepsis - complete with evidence for microbiological confirmation and independent adjudication of the sepsis diagnosis - were also recorded. Since immune cell effector function may change over the course of sepsis, we also recorded details of the timing of initial and subsequent blood samples, and the reason for blood sampling itself. Given that a recent report detected differences in genomic markers of inflammation that associate with survival within the first 24 h of intensive care admission [7], we assessed whether samples were obtained within, or beyond, this 24-h window. Since several commonly used therapies used in intensive care medicine exhibit immune modulating effects, we also recorded whether common immunomodulatory agents including antibiotics [8], glucocorticoids [9] and sedative agents [10] were documented. Reporting of pre-existing immunosuppressive or malignant disease - or their specific exclusion - was also recorded.

#### Study aims

The specific aims of each study were recorded with regard to the experimental context and primary conclusion. The context within which each of the three functional assays was studied was classified as: Pathophysiological - observational mechanistic studies detailing evolution of the assay response in clinical samples; Experimental - use of patient samples for more detailed experimental investigations beyond the assay itself; Clinical outcome - correlation of outcome measure with assay response; Biomarker comparison - correlation of alternative assay with functional assay.

### Laboratory samples

We recorded whether an *a priori* power analysis had been performed to determine the number of subjects/controls needed to refute the primary hypothesis. Sample timing and key aspects of experimental technique were compared between sepsis and control subjects. Associations made between immune cell function and clinical outcome were noted.

### Statistics

Data are presented as mean ± SD, or median (interquartile range). Age data in primary studies was used to construct 95% confidence intervals in order to assess whether differences existed between control and study populations (NCSS 8, Kaysville, UT, USA).

## Results

**Figure 1:**
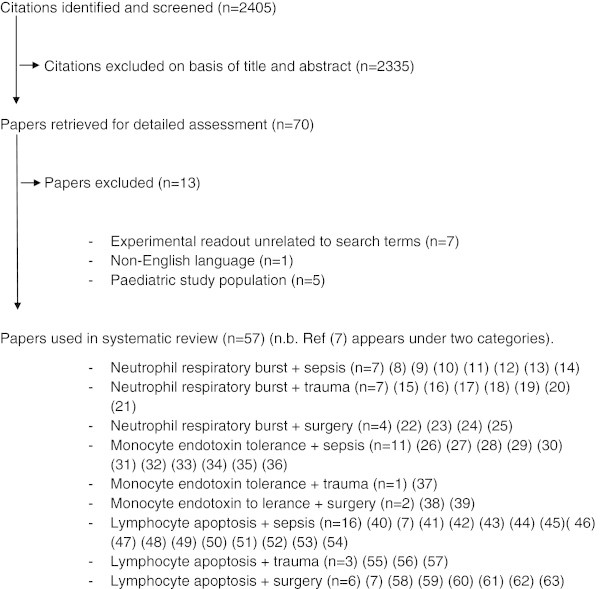
**Flow diagram illustrating study identification and inclusion** [11–66].

Fifty-seven eligible studies were identified, as summarised in Figure 1. Data is displayed into 3 tables for each immune assay, titled "Principal features of studies" (Tables 1, 2 and 3), "Demographic information" (Tables 4, 5 and 6) and "Experimental conduct and exclusion criteria" (Tables 7, 8 and 9).

**Table 1:** **Principal features of neutrophil respiratory burst studies**

Author	Study population	Subjects ( ***n*** )	Control population	Control ( ***n*** )	Experimental context	Outcome measure correlated with immune readout
Santos [12]	Sepsis	49	Healthy volunteer	19	Clinical outcome	Yes
Paunel-Gorgulu [19]	Trauma	7	Healthy volunteer	6	Experimental	No
Bruns [13]	Sepsis (cirrhotics)	45	Healthy volunteer and cohort	9 and 39	Pathophysiological	No
Shih [20]	Trauma	32	Healthy volunteer	Not provided	Biomarker comparison	Yes
Kasten [21]	Trauma	3	Healthy volunteer	3	Pathophysiological	No
Valente [22]	Trauma	24	Healthy volunteer	11	Pathophysiological	No
Kawasaki [26]	Elective surgery	20 (10,10)	Cohort	20	Pathophysiological	No
Frohlich [27]	Elective surgery	20	Cohort	20	Experimental	No
Martins [14]	Sepsis	16	Healthy volunteer	16	Pathophysiological	Yes
Barth [15]	Sepsis	27	Healthy volunteer	11	Biomarker comparison	No
Mariano [16]	Sepsis (renal replacement therapy)	7	Haemodialysis patients	10	Pathophysiological	No
Quaid [23]	Trauma	7	Healthy volunteer	Not provided	Pathophysiological	No
Wiezer [28]	Elective surgery	22 (6,6,10)	Cohort	22	Pathophysiological/experimental	Yes
Ahmed [17]	Sepsis	32	Healthy volunteer	17	Pathophysiological	No
Shih [29]	Trauma/surgery	18	Cohort and healthy volunteer	18	Pathophysiological	No
Ertel [24]	Trauma	10 (5,5)	Elective surgery	10	Pathophysiological	No
Ogura [25]	Trauma	24 (7 infected)	Cohort and healthy volunteer	24 and 15	Pathophysiological	Yes
Pascual [18]	Sepsis	23	Elective surgery	23	Pathophysiological/experimental	No

*Subjects*: values within brackets refer to subgroups within the study.

**Table 2:** **Principal features of monocyte tolerance studies**

Author	Study population	Subjects ( ***n*** )	Control population	Controls ( ***n*** )	Experimental context	Outcome measure correlated with immune readout
Liu [30]	Sepsis	2	Healthy volunteer	2	Experimental	No
Buttenschoen [41]	Elective surgery	20	Cohort	20	Pathophysiological	No
Pachot [31]	Sepsis	47	Healthy volunteer	21	Pathophysiological	Yes
West [32]	Sepsis	7	Healthy volunteer, elective surgery and SIRS	16, 5 and 4	Pathophysiological	No
Harter [33]	Sepsis	21	Healthy volunteer	12	Pathophysiological	No
Flohe [40]	Surgery in trauma patients	16	Healthy volunteer	12	Pathophysiological	No
Escoll [34]	Sepsis	3 (5)	Healthy volunteer	3	Pathophysiological	No
Heagy [39]	ICU patients (sepsis)	62	Healthy volunteer	15	Clinical outcome	Yes
Calvano [35]	Sepsis	18 (10)	Healthy volunteer	15 (6)	Pathophysiological	No
Sfeir [36]	Sepsis	10	Healthy volunteer	10	Pathophysiological	No
Kawasaki [42]	Elective surgery	20	Cohort	20	Pathophysiological	No
Heagy [37]	Sepsis	58	Healthy volunteer	14	Clinical outcome	Yes
Bergmann [38]	Sepsis	30 (2)	Healthy volunteer	12	Pathophysiological	No

*Subjects*/*controls*: numbers in brackets refer to subgroups within study.

**Table 3:** **Principal features of lymphocyte apoptosis studies**

Author	Study population	Subjects ( ***n*** )	Control population	Controls ( ***n*** )	Experimental context	Outcome measure correlated with immune readout
Roger [43]	Sepsis	48	Healthy volunteer	15	Pathophysiological	No
Bandyopadhyay [58]	Trauma	113	Healthy volunteer	?	Pathophysiological	No
White [11]	Sepsis	60	Gram negative infection and healthy volunteer	15 and 20	Pathophysiological	Yes
White [11]	Elective surgery (infective complications)	19	Cohort	41	“	“
Zhang [44]	Sepsis	19	Healthy volunteer	22	Pathophysiological	No
Guignant [45]	Sepsis	64	Healthy volunteer	49	Pathophysiological	No
Vaki [46]	Sepsis	48 (68)	Healthy volunteer	20	Pathophysiological	No
Slotwinski [62]	Elective surgery	50 (26, 24)	Cohort	50	Experimental/clinical outcome	No
Gogos [47]	Sepsis	PN 183, CAP 97, IA 100, PB 61, HAP 64	N/A		Pathophysiological	Yes
Hoogerwerf [48]	Sepsis	16	Healthy volunteer	24	Pathophysiological	No
Yousef [49]	Sepsis	32	SIRS and without SIRS	35/33	Patient outcome	Yes
Turrel-Davin [50]	Sepsis	13	Healthy volunteer	15	Biomarker comparison	No
Pelekanou [51]	Sepsis	VAP 36	Other infections	32	Pathophysiology	No
Papadima [61]	Elective surgery	40 (21, 19)	Cohort	40	Pathophysiological	No
Delogu [52]	Sepsis	16	?‘individuals’		Pathophysiological	No
Weber [53]	Sepsis	16	Non-infected ICU and healthy volunteer	10 and 11	Pathophysiological	No
Roth [54]	Sepsis	15	Healthy volunteer	20	Pathophysiological	No
Le Tulzo [55]	Sepsis	47 (25, 23)	SIRS and healthy volunteer	7 and 25	Pathophysiological/clinical outcome	Yes
Hotchkiss [56]	Sepsis	27 (FC 5) (3 intraop, 24 autopsy)	Critically ill non-septic and trauma	16 and 25 (FC 6) (3 prospective, 13 retrospective)	Pathophysiological	No
Delogu [63]	Elective surgery	18	Cohort	18	Pathophysiological	No
Pellegrini [59]	Trauma	17 (+13 burns)	Healthy volunteer	17	Clinical outcome/pathophysiological	(Correlate to MODS)
Delogu [64]	Surgical	15	Healthy volunteer	10	Pathophysiological/patient outcome	Yes
Hotchkiss [60]	Trauma	10	Elective surgery	6 (all prospective)	Pathophysiological	No
Hotchkiss [57]	Sepsis	20	Non septic prospective/non-septic retrospective/prospective trauma splenectomy/ prospective colectomy/retrospective colectomy	1/9/6/2/8	Pathophysiological	No
Sasajima [65]	Elective surgery	16 (11, 5)	Cohort	16	Pathophysiological	No
Sugimoto [66]	Elective surgery	10 (5, 5)	Cohort	10	Pathophysiological	No

**Table 4:** **Demographic information of neutrophil respiratory burst studies**

Author	Age			Gender (%male)			Subject ethnicity detailed	Severity of subject disease			Subject drug exposure documentation		
	Subjects	Controls	Statistical test result	Subjects	Controls	Statistical test result		Index	Score	No. Groups	Sedatives	Antibiotics	Steroids
Santos [12]	60 ± 17	55.3 ± 18	N	57	53	N	N	APACHE II	17 (4 to 30)	3	N	N	N
Gorgulu [19]	46 ± 4	33 ± 2	N* (*p* < 0.001)	74	59	N	N	Mortality	9%	1	N	N	N
Bruns [13]	58 (40 to 80)	45 (37 to 82); 58 (?)	0.437	82	73/48	0.341	N	-		1	N	N	N
Shih [20]	33 ± 14	?	N	66	?	N	N	ISS	23	2	N	N	N
Kasten [21]	36 ± 2	38 ± 2	*p* > 0.05	100	100	*p* > 0.05	N	ISS	23	1	N	N	N
Valente [22]	75	>65	N	46	?	N	N	ISS	15.00	1	N	N	N
Kawasaki [26]	52 ± 4; 54 ± 4	N/A	N	70	70	*p* > 0.05	N	ASA	I to II	2	Y	N	N
Frohlich [27]	66 ± 10; 69 ± 6	N/A	N	40	20	N	N	ASA	I	2	Y *t*	Y *t*	Y *t*
Martins [14]	50 ± 21	31 ± 6	N* (*p* = 0.0011)	?	?	N	N	Mortality	38%	2	N	N	N
Barth [15]	N/S (36 to 82)	24 (22 to 50)	N	60	36	N	N	Mortality	37%	1	N	N	N
Mariano [16]	67 ± 4	?	N	?	?	N	N	-		1	N	N	N
Quaid [23]	37 (20 to 71)	?	N	?	?	N	N	ISS	24 (17 to 34)		N	N	N
Wiezer [28]	57 ± 3; 62 ± 2; 58 ± 5	?	N	83, 66, 70		N	N	APACHE III	Graphs (no difference)	3	N	N	N
Ahmed [17]	55 ± 6	36 ± 16	N* (p < 0.0001)	46	?	N	N	APACHE II	20 ± 1	1	N	N	N
Shih [29]	42 ± 19	N/S	N	55	?	N	N	ISS	26 ± 7.2	3	N	N	N
Ertel [24]	N/S	?	N	?	?	N	N	AIS	Head 4.5 ± 0.2, Chest 4.1 ± 0.1	1	N	N	N
Ogura [25]	40 ± 19	35 ± 6	N	75	?	N	N	ISS	31 ± 10	2	N	N	N
Pascual [18]	59 (27 to 81)	45 (27 to 81)	*p* > 0.05	51	43	N	N	Mortality	21%	1	N	N	Y *t*

*Age*: N/S, not summarised (tabulated data for every patient provided); question mark (?), not provided within the manuscript; N/A, not applicable. Statistical test result: N, not reported; N*, not reported but we identified the significant *p* value from the original manuscript data. *Severity of subject disease*: The average clinical severity score of subjects with an index of spread listed in brackets. The number of severity groups which subjects were divided into is listed. ISS/AIS, Injury Severity Score/Abbreviated Injury Severity Score [87]; ASA, American Society of Anesthesiologists Physical Status Classification System [85]; APACHE II: Acute Physiology and Chronic Health Evaluation II [83], APACHE III, Acute Physiology and Chronic Health Evaluation III [84]. Subject drug use detailed: whether patient exposure to known immunomodulating drugs was documented. A‘*t*’ signifies that the timing of the drug administration in relation to blood sampling was clear from the study methodology.

**Table 5:** **Demographic information of monocyte tolerance studies**

Author	Age			Gender (%male)			Subject ethnicity	Severity of subject disease			Subject drug exposure documentation		
	Subjects	Controls	Statistical test result	Subjects	Controls	Statistical test result		Index	Score	No. of groups	Sedatives	Antibiotics	Steroids
Liu [30]	?	?	N	?	?	N	N	?	?	1	N	N	N
Buttenschoen [41]	56 (33 to 88)	N/A	N	70	N/A	N	N	?	?	n/a	N	N	N
Pachot [31]	68 (54 to 76)	51 (42 to 65)	N	62	52	N	N	SAPS II	51 (±5)	2	N	N	N
West [32]	N/S	N/S	N	42	100; 20; 56	N	N	?	?	2	N	N	N
Harter [33]	48 ± 20	'Comparable’	N	71	12	N	N	APACHE II	13 ± 6	1	N”	N	N
Flohe [40]	47 ± 18	37 ± 14	N	68	50	N	N	ISS	39 ± 9	1	N	N	N
Escoll [34]	51 ± 12	49 ± 12	N	?	?	N	N	?	?	1	N	N	N
Heagy [39]	49 ± 3; 44 ± 8	?	N	?	?	N	N	Mortality	20%, 9.6%	2	N	N	N
Calvano [35]	60; 61	58	N	66; 66	66	N	N	?	?	2	N	N	Y*t*
Sfeir [36]	63 ± 3	50 ± 7	N* (*p* < 0.0001)	80	50	N	N	APACHE II	27 ± 5	1	N	N	N
Kawasaki [42]	?	N/A	N	?	N/A	N	N	ASA	I to II	1	N	N	N
Heagy [37]	49 ± 21	?	N	66	?	N	N	?	?	4	N	N	N
Bergmann [38]	60; 51	32	N	?	?	N	N	MODS	15 ± 1, 7 ± 1	2	N	N	N

*Age*: N/S, not summarised (tabulated data for every patient provided); question mark (?), not provided within the manuscript; N/A, not applicable. Statistical test result: N, not reported; N*, not reported but we identified the significant *p* value from the original manuscript data. *Severity of subject disease*: The average clinical severity score of subjects with an index of spread listed in brackets. The number of severity groups which subjects were divided into is listed. ISS/AIS, Injury Severity Score/Abbreviated Injury Severity Score [87]; ASA, American Society of Anesthesiologists Physical Status Classification System [85]; APACHE II: Acute Physiology and Chronic Health Evaluation II [83], APACHE III, Acute Physiology and Chronic Health Evaluation III [84]. Subject drug use detailed: whether patient exposure to known immunomodulating drugs was documented. A‘*t*’ signifies that the timing of the drug administration in relation to blood sampling was clear from the study methodology.

**Table 6:** **Demographic information of lymphocyte apoptosis studies**

Author	Age			Gender (%male)			Ethnicity	Severity of subject disease			Subject drug exposure documentation		
	Subjects	Controls	Statistical test result	Subjects	Controls	Statistical test result		Index	Score	No. of groups	Sedatives	Antibiotics	Steroids
Roger [43]	63 (37 to 82)	55 (37 to 5)	0.04	50	43	0.76	N	SAPS II	55 (12 to 92)	2	N	Y *t*	Y *t*
Bandyopadhyay [58]	?	'Matched’	N	?	'Matched’	N	N	APACHE	>21	1	N	N	N
White [11]	54 (72 to 80)	Bacteraemia: 73 (70 to 82)	>0.05	52	Bacteraemia 40	>0.05	Y	APACHE	25 (21 to 28)	2	N	N	N
White [11]	64 ± 2	65 ± 1	0.74	68	70	0.86	N			2	N	N	N
Zhang [44]	58 ± 4	59 ± 4	N	52	50	N	N	APACHE II	26 ± 3	1	N	Y *t*	Y *t*
Guignant [45]	63 (54 to 73)	?	N	68	N	N		SAPS II	53(39 to 64)	1	N	N	Y *t*
Vaki [46]	71 ± 2	?	N	54	?	N	N	APACHE II	20 ± 9	1 (3)	N	N	N
Slotwinski [62]	62 ± 9; 63 ± 9	-	N	5, 50	-	N	N	TNM	?	1	N	Y *t*	N
Gogos [47]	67 ± 17; 68 ± 20; 54 ± 25; 64 ± 16		*P* < 0.0001	52, 62, 57, 67, 64		*P* = 0.011	N	APACHE II	12 ± 7; 16 ± 9; 13 ± 8; 18 ± 8; 20 ± 5	3	N	N	N
Hoogerwerf [48]	57 ± 5,	66 ± 5	N* (*p* < 0.0001)	63	50	N	N	APACHE II	19 ± 2	1	N	N	N
Yousef [49]	44 ± 9	45 ± 9, 44 ± 10	N	59	60, 57	N	N	SOFA	12 (7 to 14)	3 (5)	N	N	N
Turrel-Davin [50]	60 ± 4	'Age matched’	N	63	'Sex matched’	N	N	SAPS II	51 ± 3	1	N	N	Y
Pelekanou [51]	69 ± 16	64 ± 20	0.099	64	43	0.300	N	APACHE II	18 ± 4; 15 ± 5	1	N	N	Y
Papadima [61]	66 ± 7; 67 ± 10		0.8	85, 47		0.54	N	ASA	I to II	1	Y *t*	Y *t*	Y *t*
Delogu [52]	?	?	N	?	?	N	N	?	?	1	N	N	N
Weber [53]	56 ± 4	61 ± 5,?	>0.05	68, 80	?	N	N	SAPS II	26 ± 2	1	N	N	Y
Roth [54]	56 ± 6	52 ± 14	N	66	'Matched’	N	N	APACHE	N/S	1	N	N	N
Le Tulzo [55]	55 ± 4; 64 ± 4	72 ± 4; 55 ± 4	N* (*p* < 0.0001)	?	?	N	N	SAPS II	33 ± 3; 58 ± 4	2	N	N	N
Hotchkiss [56]	N/S	N/S	N	59	56, ?	N	N	-		1	N	N	Y
Delogu [63]	47 ± 17	'Matched’	N	?	'Matched’	N	N	ASA	I to II	1	Y	N	Y *t*
Pellegrini [59]	44 (20–83)	(18 to 60)	N	?	?	N	N	ISS	25 (9 to 59)	1	N	N	N
Delogu [64]	?	'Matched’	N	?	'Matched’	N	N	ASA	I to II	1	N	N	Y *t*
Hotchkiss [60]	18 to 46	?	N	90	?	N	N	ISS	N/S (9 to 50)	1	N	N	N
Hotchkiss [57]	N/S	N/S	N	65	?	N	N	-		1	N	N	Y
Sasajima [65]	62 (55 to 74); 49(37 to 58)		N		?	N	N	?	?	1	N	N	N
Sugimoto [66]	N/S		N	50		N	N	?	?	1	N	N	Y *t*

*Age*: N/S, not summarised (tabulated data for every patient provided); question mark (?), not provided within the manuscript; N/A, not applicable. Statistical test result: N, not reported; N*, not reported but we identified the significant *p* value from the original manuscript data. *Severity of subject disease*: The average clinical severity score of subjects with an index of spread listed in brackets. The number of severity groups which subjects were divided into is listed. ISS/AIS, Injury Severity Score/Abbreviated Injury Severity Score [87]; ASA, American Society of Anesthesiologists Physical Status Classification System [85]; APACHE II: Acute Physiology and Chronic Health Evaluation II [83], APACHE III, Acute Physiology and Chronic Health Evaluation III [84]. Subject drug use detailed: whether patient exposure to known immunomodulating drugs was documented. A‘*t*’ signifies that the timing of the drug administration in relation to blood sampling was clear from the study methodology.‘Matched’, paper provided no details but stated the control population was matched to the study population.

**Table 7:** **Experimental conduct and exclusion criteria of neutrophil respiratory burst studies**

Author	Study population	Sample timing		Definition of sepsis	Microbiology results provided	Independent adjudication of sepsis diagnosis	Exclusion criteria immunosuppressive disease	Exclusion criteria malignancy	Primary conclusion of study (in relation to neutrophil respiratory burst)
		Time of first sample	No. samples (time span)						
Santos [12]	Sepsis	72 h (Dx sepsis); 48 h (organ failure); onset of septic shock	2 (7 days)	1 A,B,C	N	N	Y	Y	Reactive oxygen species production by neutrophils is increased in sepsis, and [it] is associated with poor outcome
Gorgulu [19]	Trauma	24 h (Hosp Adm)	1	2 A,B,C	N	N	Y	N	Fas stimulation of septic neutrophils promotes apoptosis and inhibits functionality, partially by non-apoptotic signalling
Bruns [13]	Sepsis (cirrhotics)	24 h (Hosp Adm)	1	5	Y	N	Y	N	[Within cirrhotic patients] augmented neutrophil ROS release in response to *E. coli*…becomes exhausted in the presence of infection
Shih [20]	Trauma	24 h (Hosp Adm)	2 (3 days)	N	N	N	Y	Y	Plasma migration inhibitory factor is one of the important factors responsible for early neutrophil activation
Kasten [21]	Trauma	48 to 72 h (Post-trauma)	1	N	N	N	Y	N	Following trauma, there are concurrent and divergent immunological responses…hyper-inflammatory response by the innate arm…and hypo-inflammatory response by the adaptive arm
Valente [22]	Trauma	48 h (Hosp Adm)	3 (5 days)	N	N	N	Y	N	Injury results in differences in innate immune function in the elderly when compared with controls
Kawasaki [26]	Elective surgery	Pre-insult	5 (4 days)	N	N	N	Y	N	The innate immune system is suppressed from the early period of upper abdominal surgery
Frohlich [27]	Elective surgery	Pre-insult	2 (end of anaesth)	N	n/a	n/a	Y	Y	[This study demonstrates] suppression of neutrophil function by propofol *in vitro* [but not] *in vivo*
Martins [14]	Sepsis	48 h (ICU Adm)	1	1 B,C	Y	N	Y	Y	Neutrophil function is enhanced in patients with sepsis
Barth [15]	Sepsis	?	6 (5 days)	1C (>4d)	Y	N	N	N	Endogenous G-CSF increases neutrophil function in patients with severe sepsis and septic shock
Mariano [16]	Sepsis (renal replacement therapy)	?	4 (1 day)	1, B,D	N	N	N	N	Sera from septic patients [demonstrate] an enhanced priming activity on neutrophils [that is] reduced by ultrafiltration
Quaid [23]	Trauma	24 h (Hosp Adm)	1	N	N	N	N	N	[After severe trauma] IL-8 and GROα lose the ability to regulate the TNFα induced respiratory burst
Wiezer [28]	Elective surgery	Pre-insult	5 (7 days)	“clinical criteria”	N	N	Y	N	Patients undergoing liver resection have an increased activation of leukocytes compared with other major abdominal surgery [that is partially reversed] by endotoxin neutralisation…with rBPI_21_
Ahmed [17]	Sepsis	72 h (Proof of infection)	1	1 A,B	Y	Y	Y	Y	Septic patients deliver fewer neutrophils to secondary inflammatory sites
Shih [29]	Trauma/Surgery	24 h (Hosp adm)	3+ (7 days)	1 A,B,C	N	N	Y	Y	Surgery after [trauma] has no effect on the priming of neutrophils
Ertel [24]	Trauma	24 h (Hosp adm)	2 (3 days)	N	N	N	Y	N	Severe trauma stimulates acute-phase priming in neutrophils
Ogura [25]	Trauma	24 h (Post-trauma)	4 + 1 (21 days)	2 A B C	Y	N	N	N	Severe trauma stimulates acute-phase priming in neutrophils
Pascual [18]	Sepsis	24 h (ICU adm)	1	1 A C	Y	N	N	N	Plasma of septic patients may have a profound effect on neutrophil response [and] differentiates between sepsis and non-sepsis samples

*Sample timing*: Were control samples taken at the same time point after the inflammatory stimulus as subject samples? When was the first sample taken from the subject? How many samples were taken for each subject in total and over what time span? *Sepsis criteria*: The criteria used to enrol subjects into the study. Where subgroups of these criteria were used (e.g. septic shock) these are detailed. 0, not stated; 1, ACCP/SCCM 1992 Consensus Conference [73]; 2, ACCP/SCCM Consensus Conference 2001 [74]; 3, SSC Consensus Conference 2008 [75]; 4, CDC NNIC [86]; 5, Microbiology and clinical assessment; 6, Postmortem identification of infection; N, infection not considered; question mark (?), criteria not described. Sepsis severity groups enrolled: A = sepsis, B = severe sepsis, C = septic shock, D = acute renal failure, E = SIRS. *Microbiology documentation*: Were causative organisms clearly isolated and identified? Were additional steps taken to define whether the subject had sepsis beyond the initial clinical diagnosis, i.e. retrospective review of the case in light of subsequent information?

**Table 8:** **Experimental conduct and exclusion criteria of monocyte tolerance studies**

Author	Study population	Sample timing		Definition of sepsis	Microbiology results provided	Independent adjudication of sepsis diagnosis	Exclusion criteria immunosuppressive disease	Exclusion criteria malignancy	Primary conclusion of study (in relation to monocyte endotoxin tolerance)
		Time of first sample	No. of samples (time span)						
Liu [30]	Sepsis	?	1	? B C	N	N	N	N	TLR4 stimulation and human sepsis activate pathways that couple NAD^+^ and its sensor SIRT1 with epigenetic reprogramming
Buttenschoen [41]	Elective surgery	Pre-insult	4 (2 days)	N	N	N	Y	N	Cytokine liberation of mononuclear cells suggests a state of postoperative endotoxin tolerance
Pachot [31]	Sepsis	72 h (onset sep shock)	2	1C	Y	Y	N	N	CX3CR1 expression [is] severely down-regulated in [septic] monocytes and associated with lack of functionality
West [32]	Sepsis	24 h (ICU adm)	1	1 A, E	Y	N	N	N	Leukocytes of septic patients, but not SIRS, show LPS tolerance
Harter [33]	Sepsis	?	1	1 A B C	Y	Y	N	N	Endotoxin tolerance in septic patients does not depend solely on TLR-2 or TLR-4 expression
Flohe [40]	Surgery in trauma patients	48 h (ICU adm)	Mon, Thu.	1 A B C	Y	N	Y	Y	Initial trauma [and] major secondary surgery cause suppression of immune functions, whereas minor surgery does not
Escoll [34]	Sepsis	48 h (onset sepsis)	1	1 A	Y	Y	Y	Y	Monocytes from septic patients rapidly express IRAK-M mRNA when stimulated with LPS *ex vivo* [unlike healthy volunteers]
Heagy [39]	ICU patients (sepsis)	72 h (ICU adm)	1	5	N	Y	N	N	ICU patients with…endotoxin tolerance have significantly poorer clinical outcomes
Calvano [35]	Sepsis	?	1	1 E A	Y	N	N	N	Cellular LPS hyporesponsiveness [cannot] be ascribed to significant alterations in…cell surface LPS binding proteins
Sfeir [36]	Sepsis	24 (Sep Shock)	1	1C	Y	Y	Y	N	Monocytes from patients with septic shock exhibit persistent IL-10 release at a time when TNF-α release is down-regulated
Kawasaki [42]	Elective surgery	Pre-insult	7 (7 days)	N	N	N	Y	N	LPS responsiveness…is altered from the early period of surgery
Heagy [37]	Sepsis	72 h (ICU adm)	1	5	Y	Y	N	N	Impaired TNF release may be a manifestation of monocyte endotoxin tolerance and may be useful to diagnose sepsis
Bergmann [38]	Sepsis	?		1 B C	N	N	N	N	The altered [TNF-α release] of septic blood to catecholamines might be due to altered reactivity of leukocytes

*Sample timing*: Were control samples taken at the same time point after the inflammatory stimulus as subject samples? When was the first sample taken from the subject? How many samples were taken for each subject in total and over what time span? *Sepsis criteria*: The criteria used to enrol subjects into the study. Where subgroups of these criteria were used (e.g. septic shock) these are detailed. 0, not stated; 1, ACCP/SCCM 1992 Consensus Conference [73]; 2, ACCP/SCCM Consensus Conference 2001 [74]; 3, SSC Consensus Conference 2008 [75]; 4, CDC NNIC [86]; 5, Microbiology and clinical assessment; 6, Postmortem identification of infection; N, infection not considered; question mark (?), criteria not described. Sepsis severity groups enrolled: A = sepsis, B = severe sepsis, C = septic shock, D = acute renal failure, E = SIRS. *Microbiology documentation*: Were causative organisms clearly isolated and identified? Were additional steps taken to define whether the subject had sepsis beyond the initial clinical diagnosis, i.e. retrospective review of the case in light of subsequent information?

**Table 9:** **Experimental conduct and exclusion criteria of lymphocyte apoptosis studies**

Author	Study population	Sample timing		Definition of sepsis	Microbiology results provided	Independent adjudication of sepsis diagnosis	Exclusion criteria immunosuppressive disease	Exclusion criteria malignancy	Primary conclusion of study (in relation to lymphocyte apoptosis)
		Time of first sample	No. samples (time span)						
Roger [43]	Sepsis	Before first abs	1	3 B C	Y	Y	Y	Y	Concomitant T cell proliferation and T cell apoptosis are observed in human sepsis
Bandyopadhyay [58]	Trauma	?	Every 4 days (28 days)	N	N	N	Y	N	CD47 triggering, SHP-1 mediated NFkB suppression and elevated TRAIL levels increase…T cell apoptosis
White [11]	Sepsis	24 h (ICU adm/positive BC)	2 (7 days)	1 B C	N	Y	Y	N	Patients with infection and sepsis have deficient IL-2 and IL-7 gene expression
White [11]	Elective surgery (infective complications)	Pre-insult	3 (5 days)	4	N	Y	Y	N	
Zhang [44]	Sepsis	24 h (sep shock)	1	1C	N	N	Y	N	The expression of PD-1 on T cells [is] up regulated in septic shock
Guignant [45]	Sepsis	48 h (sep shock)	3 (10 days)	1C	Y	Y	N	Y	PD-1 related molecules may constitute a novel immunoregulatory system involved in sepsis-induced immune alterations
Vaki [46]	Sepsis	12 h (organ failure)		2 B C	Y	Y	Y	N	These findings support…the existence of an early circulating factor in severe sepsis/shock, modulating apoptosis of CD4 lymphocytes
Slotwinski [62]	Elective surgery	Pre-insult	4 (7 days)	N	N	N	Y	N	Preoperative enteral immunonutrition prevents postoperative decrease in lymphocyte subsets
Gogos [47]	Sepsis	24 h (signs of sepsis)	1	2 B C	Y	Y	Y	N	Major differences of the early statuses of innate and adaptive immune systems exist between sepsis and severe sepsis/shock in relation the underlying type of infection
Hoogerwerf [48]	Sepsis	24 h (dx sepsis)	1	2 A	Y	Y	Y	N	In patients with sepsis, alterations in apoptosis of circulating leukocytes occur in a cell-specific manner
Yousef [49]	Sepsis	?	1	1 A B C	N	N	Y	N	Percentage of apoptotic lymphocyte median values [could be] an indicator of prognosis and survival in critically ill patients
Turrel-Davin [50]	Sepsis	48 h (sep shock)	2 (5 days)	1C	Y	Y	N	N	Pro-apoptotic genes BID and FAS appear to constitute promising apoptosis markers
Pelekanou [51]	Sepsis	24 h (signs of sepsis)	1	1 2 A B C	Y	Y	Y	N	Decrease of CD-4 lymphocytes…is characteristic of sepsis arising in ventilator associated pneumonia
Papadima [61]	Elective surgery	Pre-insult	2 (1 day)	N	-		Y	Y	No alterations in lymphocyte counts [and] subpopulations [following use of epidural anaesthesia]
Delogu [52]	Sepsis	24 h (sep shock)	1	? C	Y	N	N	N	Blood caspase-1 elevated in sepsis. IL-6 correlates with apoptotic rate and caspase-9 expression in lymphocytes
Weber [53]	Sepsis	4 h (sev sepsis)	1	1 B	N	N	Y	Y	In early severe sepsis…induction of…Bim,Bid,Bak and downregulation of Bcl-2 and Bcl-xl is observed
Roth [54]	Sepsis	?	1	1 A B C	N	N	N	N	These findings strongly suggest that in septic patients Th1 T cells are selectively susceptible to apoptosis
Le Tulzo [55]	Sepsis	+ve microbiology ±3 days	2 (6 days)	1 B C E	Y	N	N	N	Lymphocyte apoptosis is rapidly increased in…septic shock…and leads to a profound and persistent lymphopaenia associated with poor outcome
Hotchkiss [56]	Sepsis	6 h (death)	1	6	Y	N	Y	N	Capsase 9 mediates profound progressive loss of B and CD4 T helper cells in [severe] sepsis
Delogu [63]	Elective surgery	Pre-insult	3 (4 days)	N	N	N	Y	Y	Surgical trauma is associated with a significant but transient increase in lymphocyte commitment to apoptosis
Pellegrini [59]	Trauma	?	2/week (until death/discharge)	N	N	N	N	N	Increased levels of apoptosis are not directly associated with negative trauma patient outcome
Delogu [64]	Surgical	Pre-insult	3 (4 days)	N	N	N	Y	Y	Surgical trauma upregulates lymphocyte death signalling factors and downregulates survival factors. Increased apoptosis of CD8+ cells maybe associated with greater risk of postsurgical infection
Hotchkiss [60]	Trauma	10 h (injury to surgery)	1	N	N	N	N	N	Focal apoptosis of intestinal epithelial and lymphoid tissues occurs extremely rapidly after injury
Hotchkiss [57]	Sepsis	6 h (death)	1	6	Y	Y	N	N	Caspase-3 mediated apoptosis causes extensive lymphocyte apoptosis in sepsis
Sasajima [65]	Elective surgery	Pre-insult	5 (7 days)	N	N	N	N	N	Transient T cell apoptosis occurs after major operations
Sugimoto [66]	Elective surgery	Pre-insult	4 (4 days)	N	N	N	N	N	Enhanced FasL expression is likely to be related to systemic inflammatory responses induced during the perioperative period

*Sample timing*: Were control samples taken at the same time point after the inflammatory stimulus as subject samples? When was the first sample taken from the subject? How many samples were taken for each subject in total and over what time span? *Sepsis criteria*: The criteria used to enrol subjects into the study. Where subgroups of these criteria were used (e.g. septic shock) these are detailed. 0, not stated; 1, ACCP/SCCM 1992 Consensus Conference [73]; 2, ACCP/SCCM Consensus Conference 2001 [74]; 3, SSC Consensus Conference 2008 [75]; 4, CDC NNIC [86]; 5, Microbiology and clinical assessment; 6, Postmortem identification of infection; N, infection not considered; question mark (?), criteria not described. Sepsis severity groups enrolled: A = sepsis, B = severe sepsis, C = septic shock, D = acute renal failure, E = SIRS. *Microbiology documentation*: Were causative organisms clearly isolated and identified? Were additional steps taken to define whether the subject had sepsis beyond the initial clinical diagnosis, i.e. retrospective review of the case in light of subsequent information?

### Source of experimental control subjects

**Figure 2:**
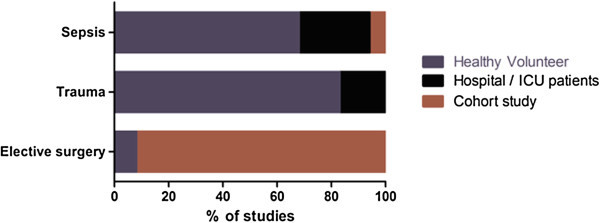
**Identification of experimental control groups.** The specific details for Hospital/ICU patients are detailed within Tables 1, 2 and 3. Within cohort study pre-insult baseline samples were taken from the study population, allowing them to act as their own experimental control.

No studies reported *a priori* power analyses based on either preceding laboratory data or *ex vivo* clinical research. The majority of studies (42/57; 74%) used case–control methodology. Control samples were obtained from healthy volunteers in (35/42; 83%), with the remainder using a variety of loosely defined clinical phenotypes (Figure 2, Tables 1, 2 and 3). The exception was elective surgical patients, where preoperative samples served as appropriate controls. Cohort methodology, where samples including controls were obtained serially from the same patient, was employed in 14/57 (25%) of studies. The majority of cohort studies were conducted in elective surgical patients (12/14; 86%).

### Age, gender and ethnicity

Advanced age is associated with progressively impaired innate and adaptive immunity [67]. Less than half of case control studies (20/42; 48%) reported the age distribution of both study and control populations. In studies where age was reported, the critically ill patients studied were often older than the control population. Female gender is associated with improved clinical outcomes following sepsis [68, 69] and increased longevity compared to males in general. Information on gender was provided in (26/42; 62%) of case–control studies. Significant variation in the incidence of sepsis has been reported according to ethnicity [70], which may reflect residual confounding or plausible biologic differences in susceptibility. However, only one study reported the ethnicity of patients.

### Co-morbidity

**Figure 3:**
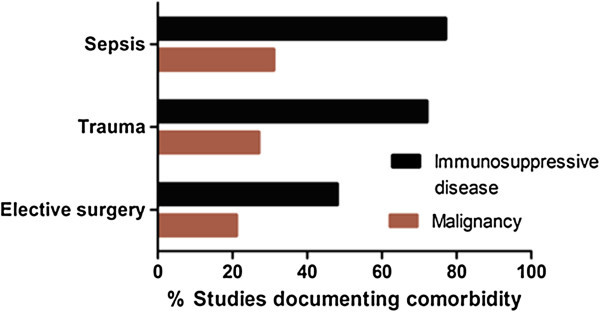
**Documentation of patients’ comorbid disease.**

Various comorbidities ranging from cardiac failure to active malignancy are associated with important deleterious alteration in effective immune function, independent of those described in sepsis [71, 72]. The majority of studies (34/57; 60%) excluded patients with overt immunosuppression while a minority (8/57; 14%) excluded those with malignancy (Figure 3).

### Clinical definition of sepsis

A high proportion of studies (26/33; 79%) defined sepsis in accordance with the ACCP/SCCM [73, 74] or Surviving Sepsis Campaign (2008 update) [75] criteria. Of those studies which used standard consensus conference criteria, (15/26, 58%) included patients with‘sepsis’, (20/26; 77%) included those with‘severe sepsis’ and (24/26, 92%) included those with‘septic shock’. In a large minority of these 26 studies (11/26; 42%), sub-categories defining sepsis were not compared separately, but combined. Immunologic studies in trauma and surgical patient samples usually did not document (18/24; 75%) whether patients developed an infection during the course of the study. In these studies, the majority (5/6) used established consensus conference criteria.

### Microbiological definitions of sepsis

Independent adjudication of the definition of sepsis used in studies was undertaken in 17/57 (30%) of studies. Since recent basic laboratory studies have demonstrated that the clinical signs/symptoms of sepsis are frequently mimicked by non-pathogenic molecules [76, 77], we sought to establish whether microbial evidence for sepsis was presented. Microbiological data were provided in 25/57 (44%).

### Severity of critical illness

A minority of studies (19/57; 33%) provided data on organ dysfunction related to sepsis severity, such as APACHE-II or SAPS II. When a severity index was used, a wide range was reported within individual studies suggesting substantial heterogeneity. In studies where mortality was reported (4/57; 7%), severity of critical illness was not reported in those patients who survived.

### Timing of experimental samples

**Figure 4:**
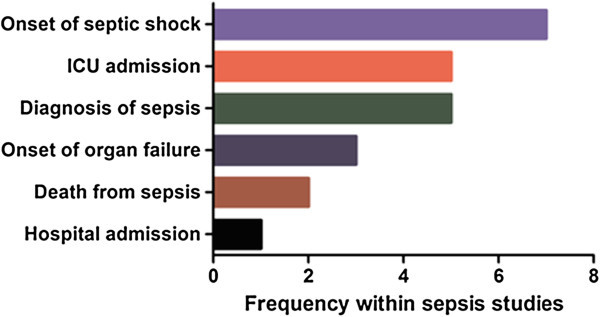
**Event trigger used for index blood sample to be taken within studies of septic patients.**

The timing of the index blood sample obtained from septic patients was described in the majority (26/33; 79%) of cases. However, the criteria for initial sampling were not comparable between studies and was most frequently defined by the severity of sepsis (Figure 4). These triggers included hospital admission (1/26), ICU admission (5/26), proof of infection (2/26), diagnosis of sepsis (5/26), onset of sepsis (14/26; 54%), onset of organ failure (3/24) and onset of septic shock (7/26) - the remaining two samples were from autopsy studies. Multiple criteria for sampling were often used and dependent upon the severity of patient illness. Approximately half of all studies (14/26; 58%) obtained an initial sample within 24 h of hospital admission. Similar patterns of sample timing were described for trauma patients. Repeat samples were often undertaken, but over highly variable intervals that were frequently not defined *a priori*. By contrast, all 12 studies undertaken in the elective surgical setting obtained preoperative control samples, with subsequent samples taken on predefined postoperative days.

### Therapies as potential confounders

**Figure 5:**
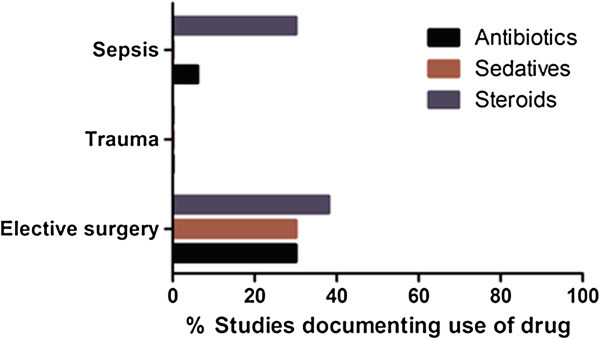
**Documentation of drug exposure of the study population.**

Commonly administered therapies in intensive care impact directly on immune function [8–10]. We assessed reporting of three of the commonest therapies with established immunomodulatory properties and found that only up to a quarter of studies documented their use (Figure 5). Specifically, these were sedative agents (4/57; 7%), antibiotics (6/57; 11%) and steroids (15/57; 26%).

### Experimental conduct and outcomes

There was no apparent relationship between the experimental context of studies and the control groups that were explored (Tables 1, 2 and 3). There are, however, clear associations between the study population studied and experimental outcome (Tables 1, 7, 2, 8, 3 and 9). For example, within the respiratory burst data, there is a consistent increase in respiratory burst identified by sepsis studies. However, since none of these studies used pre-illness samples, it is unclear if the change is a feature of sepsis, or the study population in relation to healthy volunteers. The conflicting results reported by the three surgical studies are difficult to interpret since each study uses a different burst assay, and the magnitude/type of operation varies. Similar patterns are also evident across the monocyte and lymphocyte studies.

## Discussion

This systematic review has revealed several important issues in the design and reporting of immunologic phenotype in intensive care/sepsis studies. The studies we selected are representative of the current literature, covering the past 15 years of work in three key areas of sepsis research. Following a preliminary Pubmed search, these three assays were chosen because they represent the most frequently investigation for each immune cell type. These limitations refer to the clinical aspects of the study methodology rather than specific laboratory techniques that we did not assess. These data suggest that the use of surgical patients to model critical illness may overcome several key limitations.

Defining what constitutes an adequate control sample for the immunologic study of sepsis is clearly highly challenging. Case–control studies are frequently used in sepsis research. Our review suggests that case-control studies cannot easily determine whether the observed differences in the experimental readout between the study and control groups is due to sepsis per se, or other differences between the groups including age, comorbidities and treatment interventions. Whereas cohort studies do allow pre-sepsis samples to be taken, the majority of studies are conducted in healthy volunteers free of important comorbidities (e.g. heart failure, cirrhosis) that influence both the development of, and survival from, sepsis [71]. Furthermore, age-, gender- and ethnicity-related differences in immune function are well documented [67–70], yet our data demonstrates that several key demographic details for study and control populations were frequently not reported. Finally, the presence of malignant disease - associated with immunosuppression [72] and disproportionately represented in the ICU population of most healthcare systems - was only documented in a minority of studies.

Sepsis is currently defined using clinical constructs that define syndromes, rather than use biologic and/or molecular criteria. It remains unclear whether there are biologically relevant differences between clinically defined subtypes of sepsis. In other words, changes in immunophenotype associated with progression of sepsis to severe sepsis/septic shock may merely reflect the consequences of clinical interventions and/or indirect effects on organ function that partly reflect pre-existing comorbidities. Furthermore, the specific detection of pathogens, or pathogen-associated molecular patterns, is likely to further impact on the robustness of immunophenotyping since the location and type of micro-organism both regulate host-immune responses [77, 78]. We identified only one study that specified infection site and/or a specific pathogen [34].

Critically ill patients are exposed to a range of therapeutic agents that have well-described immunologic effects. Although immunomodulation by the majority of these agents has been established *in vitro*, their role in confounding the septic immunophenotype remains unclear. Nevertheless, a myriad of off-target, immune effects have been established in pre-clinical *in vivo* models. Many antibiotics target mitochondria and eukaryotic protein synthesis [79]. Steroids exert potent pro- and anti-inflammatory properties - including inducing lymphocyte apoptosis [9]. Similarly, sedatives and analgesics exert profound effects on immune cell function [80, 81].

Our data suggest that surgical patients offer important potential advantages for mechanistic studies of sepsis. The incidence of sepsis - as defined by conventional clinical criteria - varies from 6.98% to 12.25%, depending upon the health care system and database interrogated [82]. No other patient population allows the collection of highly phenotyped data and individualised control samples prior to a defined traumatic insult. Since the volume of surgery is huge and large scale outcome data can be collected, potential limitations including comorbidities and concomitant therapies can be controlled for.

## Conclusions

We found several important limitations in clinical design associated with translational immunologic studies of human sepsis. Clinical design in mechanistic studies exploring changes in immunophenotype may contribute to the lack of translational therapeutic progress in intensive care medicine. Major elective surgery offers a potential model to overcome many of these methodological limitations.

### Take-home message

Systematic review suggests a critical re-evaluation in design of immunologic phenotyping studies conducted in intensive care.

### Tweet

Immunological investigation of septic patients presents methodological challenges that are not considered by many recent studies.

## Abbreviations

ACCP/SCCM: American College of Chest Physicians/Society of Critical Care Medicine
APACHE II: Acute Physiology and Chronic Health Evaluation [83]
APACHE III: Acute Physiology and Chronic Health Evaluation [84]
ASA: American Society of Anesthesiologists [85]
BC: Blood culture
CDC NNIS: Centre for Disease Control National Nosocomial Infections Surveillance [86]
ICU: Intensive Care Unit
ISS: Injury Severity Score [87]
MODS: Multi Organ Dysfunction Score
N/A: not applicable
N/S: not summarised
SAPS II: Simplified Acute Physiology Score
SSC: Surviving Sepsis Campaign
SIRS: Systemic Inflammatory Response Syndrome.
